# Fluid-induced acid–base variations in postoperative critically ill patients: physiological determinants and renal response

**DOI:** 10.3389/fmed.2026.1867317

**Published:** 2026-07-01

**Authors:** Francesco Zadek, Luca Zazzeron, Michele Ferrari, Davide Ottolina, Matteo Nafi, Floriana Ferrari, Eleonora Scotti, Federica Vagginelli, Marco Lattuada, Micah Liam Arthur Heldeweg, Lorenzo Giosa, Martin Krbec, Thomas Langer, Pietro Caironi

**Affiliations:** 1 Department of Medicine and Surgery, University of Milan-Bicocca, Monza, Italy; 2 Department of Anesthesia, Critical Care and Pain Medicine, Massachusetts General Hospital, Boston, MA, United States; 3 Department of Anesthesia and Intensive Care Medicine, Niguarda Ca’ Granda, Milan, Italy; 4 UO Anestesia e Rianimazione, Ospedale di Saronno, ASST Valle Olona, Saronno, Italy; 5 Department of Anesthesia and Intensive Care Medicine, IRCCS Multimedica, Sesto San Giovanni, Italy; 6 Pediatric Intensive Care Unit, Department of Anesthesiology and Intensive Care, ASST Papa Giovanni XXIII, Bergamo, Italy; 7 Department of Anesthesia, Critical Care and Emergency, Fondazione IRCCS Ca' Granda-Ospedale Maggiore Policlinico, Milan, Italy; 8 UO Anestesia e Rianimazione, Ospedale di Treviglio, ASST Bergamo Ovest, Treviglio, Italy; 9 Anaesthesia and Intensive Care Unit, E.O. Ospedali Galliera, Genoa, Italy; 10 Department of Anesthesiology, Amsterdam University Medical Centers, Amsterdam, Netherlands; 11 Department of Anaesthesiology and Intensive Care, 3rd Faculty of Medicine, Charles University and Kralovske Vinohrady University Hospital, Prague, Czechia; 12 Department of Oncology, University of Turin, Turin, Italy; 13 Department of Acute Brain and Cardiovascular Injury, Istituto di Ricerche Farmacologiche Mario Negri IRCCS, Milan, Italy

**Keywords:** acid–base, balanced solutions, bicarbonate concentration, crystalloids, fluid therapy, urine electrolyte excretion

## Abstract

**Background:**

Alterations of acid–base balance induced by intravenous fluids are primarily related to changes in plasma strong ion difference (SID_PL_) and nonvolatile weak acids. Previous studies suggest that the relationship between the SID of infused fluids (SID_INF_) and pre-infusion plasma bicarbonate (HCO_3_^−^) may act as an integrative factor influencing acid–base changes during fluid replacement. However, the contribution of unmeasured anions and renal electrolyte handling remains incompletely characterized.

**Methods:**

We conducted a prospective observational study in postoperative critically ill adults receiving intravenous fluids early after admission to intensive care unit (ICU). Fluid composition and volume were recorded. Acid–base variables, plasma and urinary electrolytes were assessed at ICU admission and study end (postoperative-day one). The average SID_INF_ and SID_INF_-HCO_3_^−^ difference were calculated. Patients were grouped by crystalloid type and SID_INF_-HCO_3_^−^ tertiles. Associations with changes in SID_PL_, standard base excess (SBE), strong ion gap (SIG), and urinary anion gap (uAG) were analyzed.

**Results:**

Fifty-seven consecutive patients were included. Patients received 3,152 ± 1,027 mL of fluids, with a slightly positive fluid balance (+802 ± 1,212 mL) over 19 [18–20] hours. SID_PL_ (38.7 ± 2.4 to 39.8 ± 2.7 mEq/L) and SBE (−1.5 ± 2.4 to 0.9 ± 2.9 mEq/L) increased (*p* < 0.001 for both), whereas SIG decreased (5.0 ± 2.8 to 3.9 ± 2.5 mEq/L; *p* < 0.001), indicating reduced unmeasured anions. Changes in SID_PL_ and SBE increased across crystalloid groups and SID_INF_-HCO_3_^−^ tertiles, with minimal SBE variation when SID_INF_-HCO_3_^−^ approximated zero. SIG changes were similar across groups. Urinary electrolyte excretion showed no quantitative association with SID_PL_ or SBE changes; however, urinary Cl^−^ decreased with increasing SID_INF_-HCO_3_^−^, consistent with renal Cl^−^ modulation.

**Conclusion:**

In postoperative critically ill patients, SID_INF_-HCO₃^−^ appears to influence fluid-induced acid–base changes, particularly during fluid replacement. Changes in unmeasured anions also contribute to SBE variations. In contrast, renal effects appear temporally limited and do not quantitatively influence plasma acid–base status over short time frames.

## Introduction

Intravenous fluids are recognized as a form of drug therapy (1, 2) that induces measurable changes in physiological conditions (3) and may exert adverse effects beyond their intended clinical use. Among these, some have been associated with worsening overall prognosis (4, 5), whereas others have been linked to specific organ dysfunctions, such as those associated with certain synthetic colloids (6, 7) and 0.9% NaCl (4, 8, 9), both of which may impair renal function. In addition, particularly in the context of fluid resuscitation and replacement, the impact of fluid composition on acid–base balance has been widely recognized (2, 10), although not extensively investigated and at times underestimated.

According to the physical–chemical approach to acid–base equilibrium proposed by Peter Stewart (11), fluid-induced acid–base alterations are primarily determined by changes in plasma strong ion difference (SID_PL_), defined as the difference between plasma concentrations of strong cations and anions and driven by the administered electrolyte composition (SID_INF_) and fluid volume, as well as by changes in the total concentration of nonvolatile weak acid (A_TOT_). Whereas A_TOT_ does not typically change during fluid maintenance, it decreases during fluid resuscitation and replacement, reflecting the absence of nonvolatile weak acids (e.g., albumin and phosphate) in commonly administered fluids (12). Our group, among others (13, 14), has recently highlighted the potential importance of pre-infusion plasma bicarbonate (HCO_3_^−^) concentration as a patient-specific factor influencing fluid-induced acid–base modifications during fluid replacement. Indeed, in a large cohort of postoperative critically ill patients (15), we retrospectively found that the difference between SID_INF_ and pre-infusion HCO_3_^−^ represents the key integrative determinant of fluid-induced acid–base alterations and identifies a reference value to which an ideal “balanced” fluid should be aligned to in order to minimize acid–base perturbations, while compensating for A_TOT_ dilution.

However, to more accurately predict fluid-induced acid–base alterations, additional factors should be considered, particularly in critically ill patients: the presence of unmeasured anions, whose changes may reflect overlapping pathological mechanisms independent of fluid administration, and the renal system, which may independently modify SID_PL_ through renal electrolyte handling and urinary excretion (16). Nonetheless, reliable data on the contribution of all determinants involved in fluid-induced acid–base alterations, including unmeasured anions, remain limited in humans. Similarly, the role of the kidney and its dynamic response to these alterations have not been clearly characterized.

We therefore designed a prospective observational study in critically ill patients admitted to a postoperative intensive care unit and receiving intravenous fluid therapy early after admission. The study aims to: (1) confirm the role of pre-infusion HCO_3_^−^ concentration, defined as the plasma level at start of fluid administration, as a key integrative factor describing acid–base changes beyond SID_INF_ and A_TOT_ variation; (2) characterize the role of changes in unmeasured anions; and (3) assess the contribution of urinary electrolyte excretion and its interaction with other determinants.

## Materials and methods

This is a single-center prospective observational study conducted at Fondazione IRCCS Ca′ Granda - Ospedale Maggiore Policlinico of Milan. The study was approved by the local Institutional Review Board (#2281, on 29/08/2007, Ethical Committee of Fondazione IRCCS Ca′ Granda - Ospedale Maggiore Policlinico, Milan, Italy), and written informed consent or deferred consent was obtained for each patient according to the national regulations. De-identification methodology was applied to patient records before analysis.

### Study population

All consecutive patients admitted to the postoperative ICU of our institution between September 2009 and June 2010, after abdominal or thoracic surgery, and having arterial and central venous catheters were included. Patients with known renal failure, liver failure with ascites, or undergoing vascular and urologic surgeries were excluded. Patients were studied from their ICU admission to 9:00 a.m. of the first postoperative day.

### Data collection

Data on patient demographics, comorbidities, medication use, reasons for admission, and type of surgery were recorded. To elucidate the effects of fluid therapy on acid–base, data concerning all types of intravenous fluid administered during the study period were strictly recorded, independent of the specific clinical indication (fluid resuscitation, replacement, or maintenance). These included volume and types of crystalloids (0.9% NaCl, Ringer’s Lactate, Rehydrating-III, Sterofundin, 5% Dextrose), colloids (6% Hydroxyethyl Starch, Polygeline), blood products, supplementary electrolytes administrations (including 8.4% NaHCO_3_), as well as crystalloid solutions employed as drug diluent or maintenance of intravenous line patency, i.e., “fluid creep” (17) (see Supplementary Table S1 for further details). In parallel, volumes of excreted fluid, including urine output, drain output and gastric residual volumes, as well as estimated insensible perspiration volume were accurately recorded (18). Finally, data on therapies applied during the study period, including diuretic therapy, were collected.

At ICU admission (postoperative day 0) and at the end of the study period (postoperative day 1), data on arterial and central venous blood gas analyses (GEM Premier 3,000, Instrumentation Laboratory, Breda, Netherlands), plasma electrolyte and albumin concentrations, and laboratory exams including metabolic, renal, liver and coagulation parameters, were obtained (COBAS c 702; Roche Diagnostics GmbH, Mannheim, Germany). In parallel, at the same time points, single-spot urine specimens were obtained for urine electrolyte assessment (Hitachi Instrument; Boehringer Mannheim GmbH, Mannheim, Germany). Finally, urine electrolyte concentrations were also measured in a representative sample derived from the total urine output collected over the study period to quantify the overall renal contribution to acid–base changes over time. Data on respiratory and hemodynamic parameters were also obtained.

### Definitions

Arterial standard base excess (SBE) was calculated as follows (19):


$$\begin{array}{c} \mathrm{SBE}\;(\frac{\text{mmol}}{L})=[\begin{array}{l} ({\mathrm{HCO}}_{3}^{-}-24.8)+(2.3\times \frac{\mathrm{Hb}}{3}+7.7)\times \\ \left(\mathrm{pH}-7.4\right) \end{array}]\times \\ \left(1-0.023\times \frac{\mathrm{Hb}}{3}\right) \end{array}$$ (1)

where HCO_3_^−^ denotes plasma bicarbonate concentration expressed in mmol/L and Hb the hemoglobin concentration expressed in mmol/L.

Plasma Strong Ion Difference (SID_PL_) was calculated as follows (11):


$$\mathrm{SI}D_{\mathrm{PL}}\;(\mathrm{mEq}/L)=[\begin{array}{l} Na^{+}+K^{+}+\\ Mg^{2+}+Ca^{2+} \end{array}]-[Cl^{-}+\text{lactat}e^{-}]$$ (2)

where Na^+^, K^+^, Mg^2+^, Ca^2+^, Cl^−^, and lactate^−^ denote sodium, potassium, magnesium, calcium, chloride, and lactate, respectively, all expressed in mEq/L.

Plasma concentration of non-volatile weak acids (A_TOT_) and their dissociated form (A^−^) were, respectively, estimated as follows (20, 21):


$$\begin{array}{c} A^{-}(\mathrm{mEq}/L)=\text{albumin}\times 10\times (0.123\times \text{arterial}\;\mathrm{pH}-0.631)+\\ P\times (0.309\times \text{arterial}\;\mathrm{pH}-0.469) \end{array}$$ (3)

where albumin was expressed as g/dL, and P indicates the plasma concentration of phosphates in mmol/L;


$$\begin{array}{l} A_{\mathrm{TOT}}(\text{mmol}/L)=A^{-}\times (1+10^{\left(\text{arterial}\;\mathrm{pH}-6.8\right)})/\\ \left(10^{\left(\text{arterial}\;\mathrm{pH}-6.8\right)}\right) \end{array}$$ (4)

*Effective* Strong Ion Difference (SID_EFF_) was calculated as follows (22, 23):


$$\mathrm{SI}D_{\mathrm{EFF}}\;(\mathrm{mEq}/L)=[{\mathrm{HCO}}_{3}^{-}]+[A^{-}]$$ (5)

In addition, the Strong Ion Gap (SIG), denoting net unmeasured anions (22), was defined as the difference between SID_PL_ and SID_EFF_:


$$\mathrm{SIG}\;(\mathrm{mEq}/L)=[\mathrm{SI}D_{\mathrm{PL}}]-[\mathrm{SI}D_{\mathrm{EFF}}]$$ (6)

The total volume of administered fluids was calculated as the sum of the volume of all crystalloids (0.9% NaCl, Ringer’s Lactate, Rehydrating-III, Sterofundin, 5% Dextrose), colloids (6% Hydroxyethyl Starch, Polygeline), blood products, supplementary electrolytes administrations, and fluid creep administered during study period. Subsequently, the average SID infused over the study period (SID_INF_) was calculated as follows:


$$\mathrm{SI}D_{\mathrm{INF}}(\mathrm{mEq}/L)=\frac{\sum (\mathrm{SI}D_{S}\times V)}{\sum V}$$ (7)

where SID_S_ denotes the *in-vivo* SID in mEq/L of each fluid administered, assuming complete metabolism of organic anions (Supplementary Table S1), and V the volume of each fluid administered during the study period, as expressed in liters. Net fluid balance over the study period was calculated as the difference between total fluid administered, including fluid creep, and the total output volume.

Mean urinary Anion Gap (uAG) was calculated as follows (24, 25):


$$\mathrm{uAG}\;(\mathrm{mEq}/L)=[\mathrm{uN}a^{+}+uK^{+}]-[\mathrm{uC}l^{-}]$$ (8)

where uNa^+^, uK^+^, and uCl^−^ denote, respectively, urine sodium, potassium, and chloride concentrations, all expressed in mEq/L, and obtained from a representative urinary sample obtained from the total urine output collected over the study period.

Finally, to investigate the impact of fluid therapy on acid–base, plasma, and urinary electrolyte concentrations, we calculated the difference (∆) in SBE, SID_PL_, and single-spot urine electrolyte concentrations and uAG assessed at the end of the study (9:00 a.m. of the first postoperative day) and at ICU admission. Overall, no imputation was applied for potentially missing data.

### Study subgroups

To first evaluate the effects of fluid therapy on acid–base balance, we considered the study population as divided by the type of crystalloid predominantly received during study period, as clinically prescribed by the attending physician: Sterofundin group, receiving mainly Sterofundin (and no Rehydrating-III); Rehydrating-III group, receiving mainly Rehydrating-III (and no Sterofundin); and miscellaneous group, receiving different types of crystalloids (including 0.9% NaCl, Sterofundin, Ringer’s Lactate, Rehydrating-III, 5% Dextrose). Subsequently, to confirm the key integrative role of the difference between SID_INF_ and pre-infusion plasma HCO_3_^−^ concentration on acid–base changes, based on the variation observed in SID_INF_ and plasma HCO_3_^−^ levels (Supplementary Figures S1, S2), we further divided the study population according to the tertile distribution of SID_INF_-HCO_3_^−^.

### Statistical analysis

Based on our previous study (15), we estimated that a sample size of 51 patients would be required to detect a minimum mean difference of 3.40 mEq/L in post-infusion BE variation across tertiles of the SID_INF_-HCO_3_^−^ difference, using a one-way ANOVA, assuming a residual standard deviation of 3.04 mEq/L, with 80% power and a two-sided *α* of 0.05. Allowing for potential non-normality, the sample size was increased by approximately 10–12%, yielding a final target of 57 patients. Data are presented as mean ± standard deviation, median [interquartile range], or frequency (percentage), as appropriate. Normality of data distribution was assessed using the Shapiro–Wilk test. Continuous variables were analyzed using the paired *t*-test, Wilcoxon signed-rank test, or one-way or two-way analysis of variance (ANOVA) with Holm-Sidak correction for multiple comparisons, as appropriate. When data were not normally distributed, the Kruskal-Wallis test with Dunn’s *post hoc* correction was applied. Categorical variables were compared using the Chi-square or Fisher’s exact test, as appropriate. A *p*-value < 0.05 was considered statistically significant. Analyses were performed using Stata statistical software (Stata Statistical Software 19.5; StataCorp, College Station, TX, United States) and SigmaPlot 15.0 (Systat Software, San Jose, CA, United States). The Strengthening the Reporting of Observational Studies in Epidemiology checklist (26) was employed (see Supplementary materials).

## Results

### Study population

A total of 57 consecutive patients (70 ± 13 years, 35% female) admitted to the postoperative ICU after major surgery were included in the study. Of these, 27 (47%) underwent thoracic surgery, 24 (42%) gastrointestinal surgery, 3 (5%) hepatic surgery, and 3 (5%) other surgery/procedures. Demographic and anamnestic data are summarized in Table 1.

**Table 1:** Demographic and clinical characteristics of the overall study population according to the type of crystalloid infused.

Characteristics	Overall population (*n* = 57)	Sterofundin (*n* = 13)	Miscellaneous (*n* = 20)	Rehydrating III (*n* = 24)	*p*-value
Age – year	70 ± 13	70 ± 13	71 ± 14	68 ± 12	0.84
Female – *n* (%)	20 (35)	5 (38)	7 (35)	8 (33)	1.00
BMI – kg/m^2^	25 ± 4	23 ± 4	26 ± 4	26 ± 4	0.04
Comorbidities					
Ischemic heart disease – *n* (%)	18 (31)	5 (38)	7 (35)	6 (25)	0.65
Hypertension – *n* (%)	30 (53)	7 (54)	12 (60)	11 (46)	0.67
Cirrhosis – *n* (%)	4 (7)	1 (8)	3 (15)	1 (4)	0.52
COPD – *n* (%)	19 (33)	4 (31)	5 (25)	10 (42)	0.52
Creatinine – mg/dL	0.82 ± 0.22	0.79 ± 0.22	0.86 ± 0.26	0.80 ± 0.18	0.56
eGFR – mL/min	86 ± 32	76 ± 22	88 ± 38	90 ± 31	0.46
Urea – mg/dL	32 ± 10	34 ± 10	29 ± 12	32 ± 9	0.48
Causes of admission – *n* (%)					0.14
Gastrointestinal surgery	24 (42)	7 (54)	9 (45)	8 (34)	
Hepatic surgery	3 (5)	1 (8)	0 (0)	2 (8)	
Thoracic surgery	27 (47)	3 (23)	10 (50)	14 (58)	
Others	3 (5)	2 (15)	1 (5)	0 (0)	
Mechanically Ventilated – *n* (%)	57 (100)	13 (100)	20 (100)	24 (100)	1.00
Duration of mechanical ventilation–hour	3 [2–4]	3 [2–3]	3 [3–4]	3 [2–4]	0.15
Study period – hours	19 [18–20]	18 [18–19]	19 [18–20]	19 [18–20]	0.30

BMI denotes body mass index; COPD, chronic obstructive pulmonary disease; eGFR, estimated glomerular filtration rate. Data are presented as mean ± standard deviation, median [interquartile range], or n (%), as appropriate. *p* values refer to one-way analysis of variance (ANOVA), Kruskal-Wallis one-way ANOVA on ranks, or Chi-square test as appropriate.

### Fluid-induced acid–base changes in the overall population

The study period lasted 19 (18–20) hours. Overall, patients received a mean fluid volume of 3,152 ± 1,027 mL, while the total fluid output was 2,350 ± 693 mL, resulting in a slightly positive fluid balance (802 ± 1,212 mL; Table 2). In parallel, although albumin concentration decreased, A_TOT_ remained unchanged, due to a significant increase in phosphate concentration. The mean SID_INF_ in the study population was 33.7 ± 13.1 mEq/L. Overall, a significant increase in SID_PL_ (from 38.7 ± 2.4 to 39.8 ± 2.7 mEq/L) and SBE (from −1.5 ± 2.4 to 0.9 ± 2.9 mmol/L, *p* < 0.001 for both; Supplementary Table S2) was observed at the end of the study period. Of note, in parallel with these changes, plasma SIG significantly decreased (from 5.0 ± 2.8 to 3.9 ± 2.5 mEq/L, *p* < 0.001), denoting a reduction in unmeasured anion concentrations. In addition, arterial PCO_2_ increased by 2.8 ± 5.8 mmHg (*p* < 0.001). Overall, arterial pH remained stable over the study period (*p* = 0.22).

**Table 2:** Fluid therapy and electrolytes administered during the study period according to the type of crystalloid infused.

Characteristics	Overall population (*n* = 57)	Sterofundin (*n* = 13)	Miscellaneous (*n* = 20)	Rehydrating III (*n* = 24)	*p*-value
Total amount of administered fluids – mL	3,152 ± 1,027	3,304 ± 1,154	3,433 ± 1,037	2,836 ± 893	0.13
Infused Solutions – mL					
Sterofundin	580 ± 1,086	2,429 ± 778	75 ± 245	0 ± 0	<0.001
Normal Saline	362 ± 728	0 ± 0	1,032 ± 913	0 ± 0	<0.001
Rehydrating III	1,412 ± 1,071	0 ± 0	1,469 ± 849	2,129 ± 729	<0.001
Others^c^	417 ± 369	490 ± 544	452 ± 315	349 ± 294	0.48
Fluid creep	381 ± 141	385 ± 151	405 ± 127	359 ± 148	0.56
Infused electrolytes^d^ – mEq					
Na^+^	422 ± 145	430 ± 140	481 ± 158	368 ± 119	0.03
K^+^	34 ± 24	24 ± 25	40 ± 21	35 ± 24	0.18
Ca^2+^	11 ± 5	14 ± 6	10 ± 6	11 ± 5	0.19
Mg^2+^	9 ± 5	7 ± 5	8 ± 5	9 ± 6	0.57
Cl ^−^	371 ± 132	408 ± 135	435 ± 131	297 ± 93	0.001
Total amount of excreted fluids – mL	2,350 ± 693	2,259 ± 786	2,314 ± 816	2,430 ± 534	0.75
Drainage	397 ± 380	246 ± 234	466 ± 468	422 ± 353	0.25
Nasogastric losses	57 ± 127	94 ± 193	66 ± 129	30 ± 67	0.33
Insensible perspiration	699 ± 173	628 ± 155	698 ± 193	739 ± 158	0.18
Diuresis	1,197 ± 612	1,291 ± 754	1,084 ± 739	1,240 ± 382	0.58
Net fluid balance – mL	802 ± 1,212	1,045 ± 1,519	1,119 ± 1,271	406 ± 868	0.11
Diuretic – *n* (%)	21 (37)	2 (15)	9 (45)	10 (42)	0.12
SID_INF_ – mEq/L	33.7 ± 13.1	20.3 ± 4.8	30.5 ± 13.2	43.6 ± 6.8^ab^	<0.001
SID_INF_ - HCO_3_^−^ – mEq/L	10.6 ± 12.3	−1.3 ± 5.1	7.5 ± 13.0	19.6 ± 6.6	<0.001

Na^+^ denotes sodium; K^+^ potassium; Ca^2+^ ionized calcium; Mg^2+^ magnesium; Cl^−^ chloride; SID_INF_ infused SID. Data are presented as mean ± standard deviation or *n* (%), as appropriate. *p* values refer to one-way analysis of variance (ANOVA), Kruskal-Wallis one-way ANOVA on ranks, or chi-square test as appropriate. a = *p* < 0.05 vs. Sterofundin; b = *p* < 0.05 vs. Miscellaneous; c = “Others” encompasses the administered volumes of other crystalloids, of colloids, and of electrolytes correction; d = the infused electrolytes balance account also for the presence of additional electrolytes supplementation.

### Influence of crystalloid composition on acid–base

Patients were grouped according to the crystalloid solution predominantly received: the Sterofundin group (*N* = 13; mean SID_INF_ 20.3 ± 4.8 mEq/L), the Miscellaneous group (*N* = 20; mean SID_INF_ 30.5 ± 13.2 mEq/L), and the Rehydrating-III group (*N* = 24; mean SID_INF_ 43.6 ± 6.8 mEq/L) (Tables 1, 2). Based on the plasma HCO_3_^−^ concentration at ICU admission, prior to fluid therapy, the corresponding SID_INF_-HCO_3_^−^ differences were −1.3 ± 5.1 mEq/L, 7.5 ± 13.0 mEq/L, and 19.6 ± 6.6 mEq/L in the Sterofundin, Miscellaneous, and Rehydrating-III groups, respectively. No differences were observed between groups in total study duration, duration of mechanical ventilation, total fluid volume administered, or net fluid balance (Tables 1, 2). Moreover, baseline acid–base parameters at ICU admission were comparable across the three groups, with the exception of SBE and pre-infusion HCO_3_^−^, both higher in the Rehydrating-III group (Table 3). Consistent with the overall study population, SBE significantly increased across the three SID_INF_ groups (Table 3). In the Sterofundin and Miscellaneous groups, the observed ΔSBE was primarily driven by a reduction in SIG (by −1.6 ± 2.7 and −1.3 ± 2.6 mEq/L, respectively; *p* = 0.03 and *p* = 0.02), as no significant modification in SID_PL_ was detected. In contrast, in the Rehydrating-III group, ΔSBE was associated with a significant increase in SID_PL_ (by 2.4 ± 2.0 mEq/L, *p* < 0.001), with no significant change in SIG. A_TOT_ dilution was similar between groups (*p* = 0.01 for time factor, two-way ANOVA; Table 3).

**Table 3:** Blood gases, acid–base parameters, and electrolyte characteristics at ICU entry and study end according to the type of crystalloid infused.

Variable	Sterofundin (*n* = 13)		Miscellaneous (*n* = 20)		Rehydrating III (*n* = 24)		*p*-value between groups	*p*-value time	Interaction *p*
	Baseline	End of study	Baseline	End of study	Baseline	End of study			
pH	7.40 ± 0.06	7.41 ± 0.03	7.40 ± 0.05	7.42 ± 0.03	7.42 ± 0.05	7.42 ± 0.03	0.18	0.20	0.43
PCO_2_ – mmHg	35.5 ± 5.4	36.4 ± 3.8	37.0 ± 4.0	38.7 ± 3.3	37.1 ± 5.7	42.0 ± 4.1*	0.02	0.002	0.07
HCO_3_^−^ – mEq/L	21.6 ± 1.6	22.9 ± 1.7	23.0 ± 2.2	25.2 ± 2.1*	24.0 ± 2.4	27.3 ± 2.9*	<0.001	<0.001	0.02
SBE – mmol/L	−3.0 ± 1.8	−1.7 ± 1.6*	−1.7 ± 2.4	0.7 ± 2.2*	−0.4 ± 2.3	2.6 ± 2.9*	<0.001	<0.001	0.06
SID_PL_ – mEq/L	38.6 ± 2.0	38.1 ± 1.4	38.3 ± 3.4	39.0 ± 2.5	39.1 ± 1.5	41.5 ± 2.4*	0.01	0.004	<0.001
SID_EFF_ – mEq/L	32.0 ± 1.6	33.1 ± 2.0	33.2 ± 3.6	35.2 ± 3.1*	34.9 ± 2.5	38.2 ± 3.3*	<0.001	<0.001	0.02
SIG – mEq/L	6.6 ± 2.7	5.0 ± 1.3*	5.1 ± 2.9	3.8 ± 2.2*	4.1 ± 2.4	3.2 ± 2.9	0.02	<0.001	0.71
Na^+^ − mEq/L	138.2 ± 2.4	138.8 ± 3.0	137.1 ± 3.6	137.9 ± 2.9	139.0 ± 2.1	139.4 ± 2.4	0.08	0.09	0.89
K^+^ − mEq/L	4.0 ± 0.4	4.2 ± 0.1	3.8 ± 0.4	4.0 ± 0.4	3.7 ± 0.3	4.0 ± 0.4*	0.04	0.001	0.70
Ca^2+^ − mEq/L	2.1 ± 0.1	2.1 ± 0.1	2.0 ± 0.1	2.0 ± 0.1	2.1 ± 0.1	2.1 ± 0.1	0.01	0.18	0.88
Mg^2+^ − mEq/L	1.5 ± 0.3	1.6 ± 0.2*	1.4 ± 0.2	1.6 ± 0.1*	1.5 ± 0.2	1.7 ± 0.2*	0.17	<0.001	0.51
Cl^−^ – mEq/L	106 ± 2	108 ± 3*	105 ± 4	105 ± 3	106 ± 2	105 ± 2.9*	0.11	0.49	0.003
Lac^−^ – mEq/L	1.0 ± 0.4	1.0 ± 0.4	1.4 ± 1.0	1.1 ± 0.8	1.0 ± 0.4	0.8 ± 0.2	0.07	0.16	0.34
A_TOT_ – mmol/L	13.0 ± 1.1	12.8 ± 1.4	12.6 ± 2.3	12.1 ± 1.6	13.5 ± 1.8	13.6 ± 1.5	0.06	0.01	0.35
Alb^−^ – mEq/L	8.5 ± 0.8	8.2 ± 1.0	8.3 ± 1.6	7.8 ± 1.3*	9.2 ± 1.4	8.8 ± 1.3*	0.04	<0.001	0.81
P^−^ – mEq/L	1.9 ± 0.3	2.1 ± 0.3*	1.7 ± 0.4	1.9 ± 0.3*	1.8 ± 0.3	2.2 ± 0.4*	0.12	<0.001	0.38
PaO_2_ – mmHg	141 ± 79	79 ± 30	167 ± 46	84 ± 23	167 ± 48	84 ± 23	0.23	<0.001	0.54
FiO_2_ – %	45 ± 10	33 ± 22	47 ± 5	25 ± 8	42 ± 8	24 ± 5	0.05	<0.001	0.19
Hb – g/dL	11.5 ± 1.4	11.6 ± 1.2	11.5 ± 1.7	10.5 ± 1.5*	11.4 ± 1.7	11.1 ± 1.9	0.52	0.10	0.14

PaCO_2_ denotes arterial partial pressure of carbon dioxide; HCO_3_^−^ bicarbonate concentration; SBE standard base excess; SID_PL_ plasma strong ion difference; SID_EFF_ effective strong ion difference; SIG strong ion gap; A_TOT_ total concentration of non-carbonic weak acids; Alb^−^ ionized concentration of albumin; P^−^ ionized concentration of phosphate; Hb hemoglobin; PaO_2_ arterial partial pressure of oxygen; and FiO_2_ fraction of inspired oxygen. Data are presented as mean ±standard deviation. *p* values refer to two-way repeated measurement analysis of variance (ANOVA) with *post-hoc* all pairwise multiple comparison procedures (Holm-Sidak correction methods). *p < 0.05 vs baseline within the same group.

### Influence of pre-infusion HCO_3_ concentrations on acid–base

**Figure 1:**
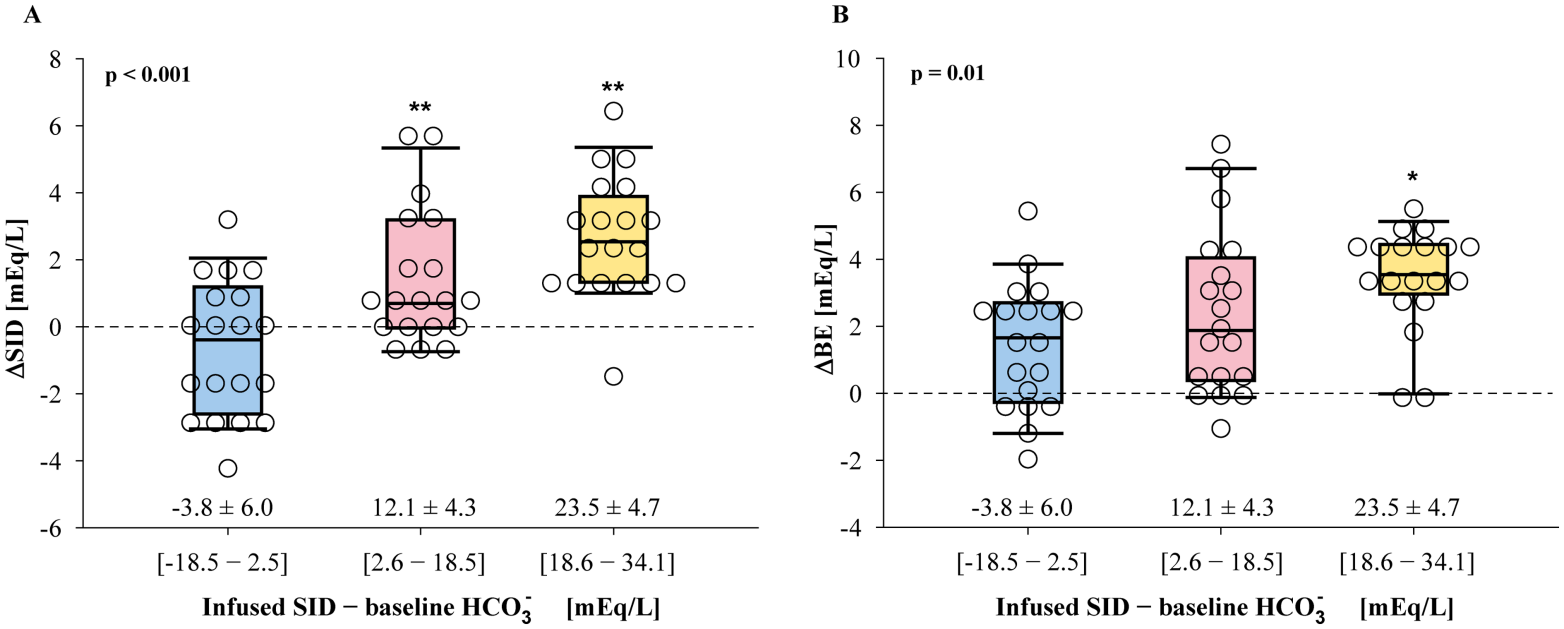
Effect of the average difference between SID infused (SID_INF_) and plasma bicarbonate concentration on plasma SID (SID_PL_) **(A)** and standard base excess (SBE) **(B)** variations during the study period, stratified by tertiles of the SID_INF_–HCO₃^−^ difference. Blue boxes represent the first tertile, red boxes the second tertile, and yellow boxes the third tertile. Variations are calculated as the difference between end-of-study and baseline values for each parameter. Data are presented as box plot (median, 25–75, whiskers indicate the 10th–90th percentiles). For each tertile, the range (minimum–maximum) of the SID_INF_–HCO₃^−^ difference is reported. *p* values refer to one-way analysis of variance (ANOVA) with *post-hoc* all pairwise multiple comparison procedures (Holm-Sidak correction methods). * *p* < 0.05; ** *p* < 0.001 vs. first tertile.

**Figure 2:**
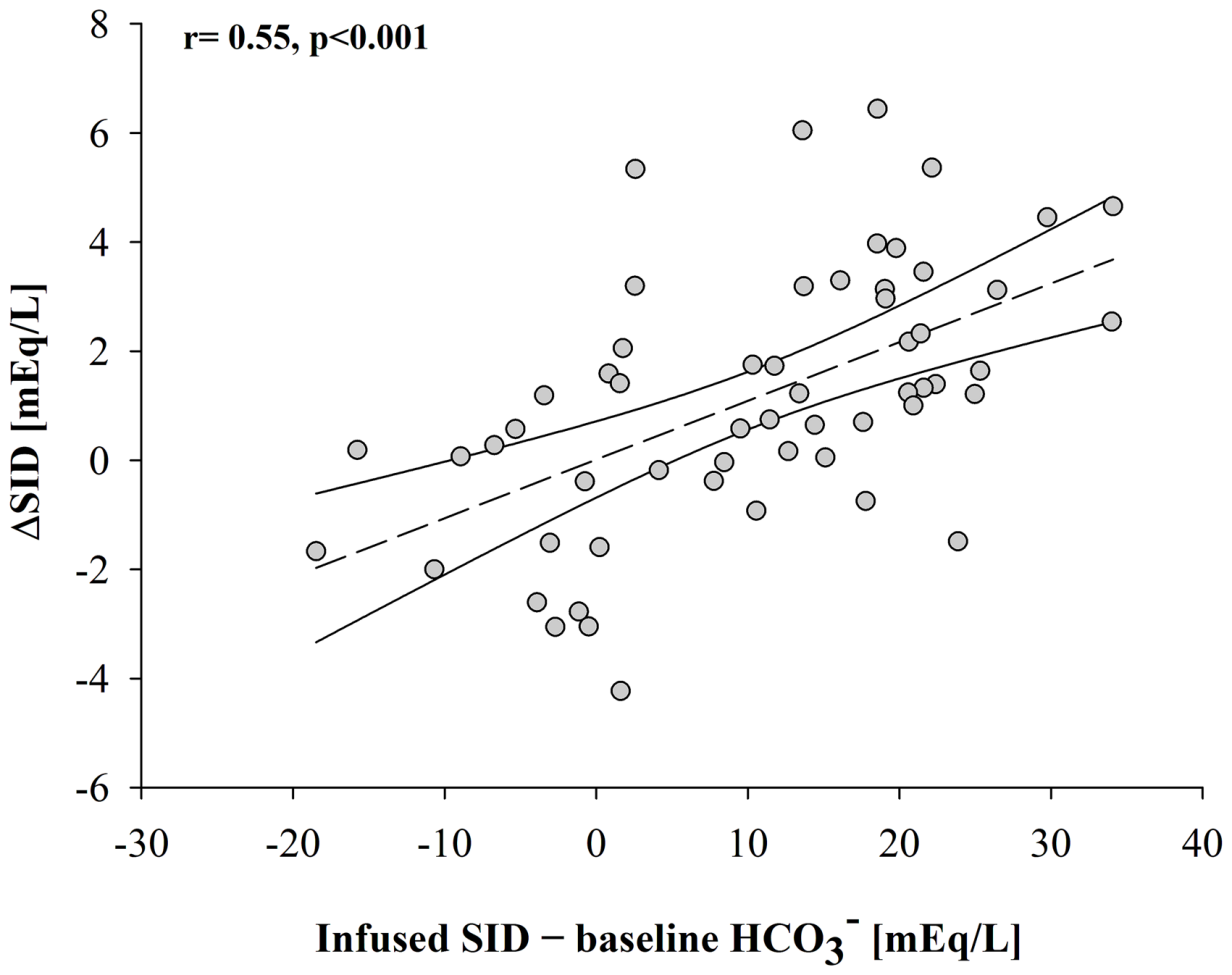
Association between the average difference between SID infused (SID_INF_) and plasma bicarbonate concentration and plasma SID (SID_PL_) difference. Pearson’s *r* = 0.55, *p* < 0.001. Model’s equation: $\text{ΔSID}=0.11\cdot ({\mathrm{SID}}_{\mathrm{INF}}-\mathrm{HC}O_{3}^{-})+0.02.$

To better characterize fluid-induced acid–base changes, the study population was stratified into tertiles according to the SID_INF_-HCO_3_^−^ difference, reflecting variability in pre-infusion plasma HCO_3_^−^ concentration (Supplementary Figure S1), with mean values of −3.8 ± 6.0 mEq/L, 12.1 ± 4.3 mEq/L, and 23.5 ± 4.7 mEq/L, respectively. At baseline, the three groups were comparable in terms of SID_PL_ and overall acid–base status, except for slight differences in SBE and pre-infusion plasma HCO_3_^−^ (Table 4). At the end of the study, after fluid administration, SID_PL_ increased significantly in the 2° and 3° SID_INF_-HCO_3_^−^ tertiles, whereas SBE increased in all groups. Overall, the magnitude of ΔSID_PL_ and ΔSBE progressively increased across SID_INF_-HCO_3_^−^ tertiles (p for interaction <0.001 and p for interaction = 0.01, respectively; Table 4 and Figure 1). Moreover, in the overall population, SID_INF_-HCO_3_^−^ difference appeared linearly associated with ΔSID_PL_ (Figure 2; Pearson’s *r* = 0.55, *p* < 0.001). In addition to these changes, SIG decreased significantly during the study period, with no differences across SID_INF_-HCO_3_^−^ tertiles (*p* for interaction = 0.35, two-way ANOVA; Table 4).

**Table 4:** Blood gases, acid–base parameters, and electrolyte characteristics at ICU admission and study end, stratified according to the tertiles of the SID_INF_–HCO₃^−^ difference.

Variable	T_1_ (*n* = 19) [−18.5–2.5]		T_2_ (*n* = 19) [2.6–18.5]		T_3_ (*n* = 19) [18.6–34.1]		*p*-value between groups	*p*-value time	Interaction *p*
	Baseline	End of study	Baseline	End of study	Baseline	End of study			
pH	7.40 ± 0.05	7.41 ± 0.03	7.42 ± 0.05	7.43 ± 0.03	7.41 ± 0.06	7.43 ± 0.03	0.06	0.23	0.93
PCO_2_ – mmHg	36.3 ± 4.8	37.6 ± 3.5	37.0 ± 4.2	40.2 ± 4.5*	36.9 ± 6.1	40.9 ± 4.5*	0.21	<0.001	0.35
HCO_3_^−^ – mEq/L	22.1 ± 1.7	23.5 ± 1.7*	23.9 ± 2.2	26.4 ± 3.1*	23.4 ± 2.7	26.8 ± 2.7*	0.002	<0.001	0.008
SBE – mmol/L	−2.6 ± 1.9	−1.2 ± 1.7*	−0.6 ± 2.4	1.8 ± 3.1*	−1.2 ± 2.7	2.2 ± 2.6*	0.001	<0.001	0.01
SID_PL_ – mEq/L	39.3 ± 2.1	38.6 ± 1.5	38.6 ± 2.9	40.0 ± 3.1*	38.2 ± 2.3	40.8 ± 2.8*	0.76	<0.001	<0.001
SID_EFF_ – mEq/L	32.6 ± 1.9	33.6 ± 2.2	34.4 ± 3.7	36.8 ± 4.2	33.9 ± 2.9	37.4 ± 3.1	0.02	<0.001	0.01
SIG – mEq/L	6.8 ± 2.4	5.0 ± 1.6*	4.2 ± 3.2	3.3 ± 2.8	4.2 ± 1.8	3.4 ± 2.6	0.004	<0.001	0.35
Na^+^ − mEq/L	138.2 ± 2.7	138.8 ± 3.0	137.7 ± 3.0	137.9 ± 2.6	138.5 ± 2.9	139.5 ± 2.4	0.34	0.08	0.63
K^+^ − mEq/L	4.0 ± 0.4	4.2 ± 0.2	3.7 ± 0.3	4.0 ± 0.3*	3.8 ± 0.4	3.9 ± 0.5	0.003	<0.001	0.36
Ca^2+^ − mEq/L	2.0 ± 0.1	2.0 ± 0.1	2.0 ± 0.1	2.0 ± 0.1	2.0 ± 0.2	2.1 ± 0.1	0.90	0.16	0.84
Mg^2+^ − mEq/L	1.5 ± 0.3	1.6 ± 0.2*	1.5 ± 0.2	1.7 ± 0.2*	1.4 ± 0.3	1.6 ± 0.2*	0.77	<0.001	0.18
Cl^−^ – mEq/L	106 ± 3	107 ± 3	105 ± 3	105 ± 3	107 ± 2	105 ± 3*	0.15	0.96	0.004
Lac^−^ – mEq/L	0.9 ± 0.4	0.9 ± 0.4	1.4 ± 1.0	1.0 ± 0.5	1.1 ± 0.4	1.0 ± 0.6	0.27	0.08	0.13
A_TOT_ – mmol/L	13.0 ± 1.3	12.6 ± 1.4	13.1 ± 2.3	12.9 ± 1.9	13.2 ± 2.0	13.1 ± 1.6	0.89	0.30	0.91
Alb^−^ – mEq/L	8.4 ± 0.9	8.0 ± 1.1	8.8 ± 1.7	8.4 ± 1.6	8.9 ± 1.5	8.5 ± 1.1*	0.48	0.001	0.98
P^−^ – mEq/L	1.9 ± 0.3	2.1 ± 0.3	1.8 ± 0.3	2.0 ± 0.3*	1.7 ± 0.4	2.1 ± 0.5*	0.28	<0.001	0.27
PaO_2_ – mmHg	151 ± 53	81 ± 30	157 ± 86	86 ± 28	171 ± 47	81 ± 21	0.53	<0.001	0.48
FiO_2_ – %	47 ± 9	30 ± 19	44 ± 5	25 ± 7	43 ± 9	24 ± 5	0.06	<0.001	0.78
Hb – g/dL	12.0 ± 1.2	11.3 ± 1.2	10.9 ± 1.9	10.7 ± 1.9	11.6 ± 1.6	11.0 ± 1.8	0.16	0.06	0.68

PaCO_2_ denotes arterial partial pressure of carbon dioxide; HCO_3_^−^ bicarbonate concentration; SBE standard base excess; SID_PL_ plasma strong ion difference; SID_EFF_ effective strong ion difference; SIG strong ion gap; A_TOT_ total concentration of non-carbonic weak acids; Alb^−^ ionized concentration of albumin; P^−^ ionized concentration of phosphate; Hb hemoglobin; PaO_2_ arterial partial pressure of oxygen; and FiO_2_ fraction of inspired oxygen. Data are presented as mean ± standard deviation. *p* values refer to two-way repeated measurement analysis of variance (ANOVA) with *post-hoc* all pairwise multiple comparison procedures (Holm-Sidak correction methods). *p < 0.05 vs baseline within the same tertile.

### Influence of urinary electrolyte excretion on acid–base

**Figure 3:**
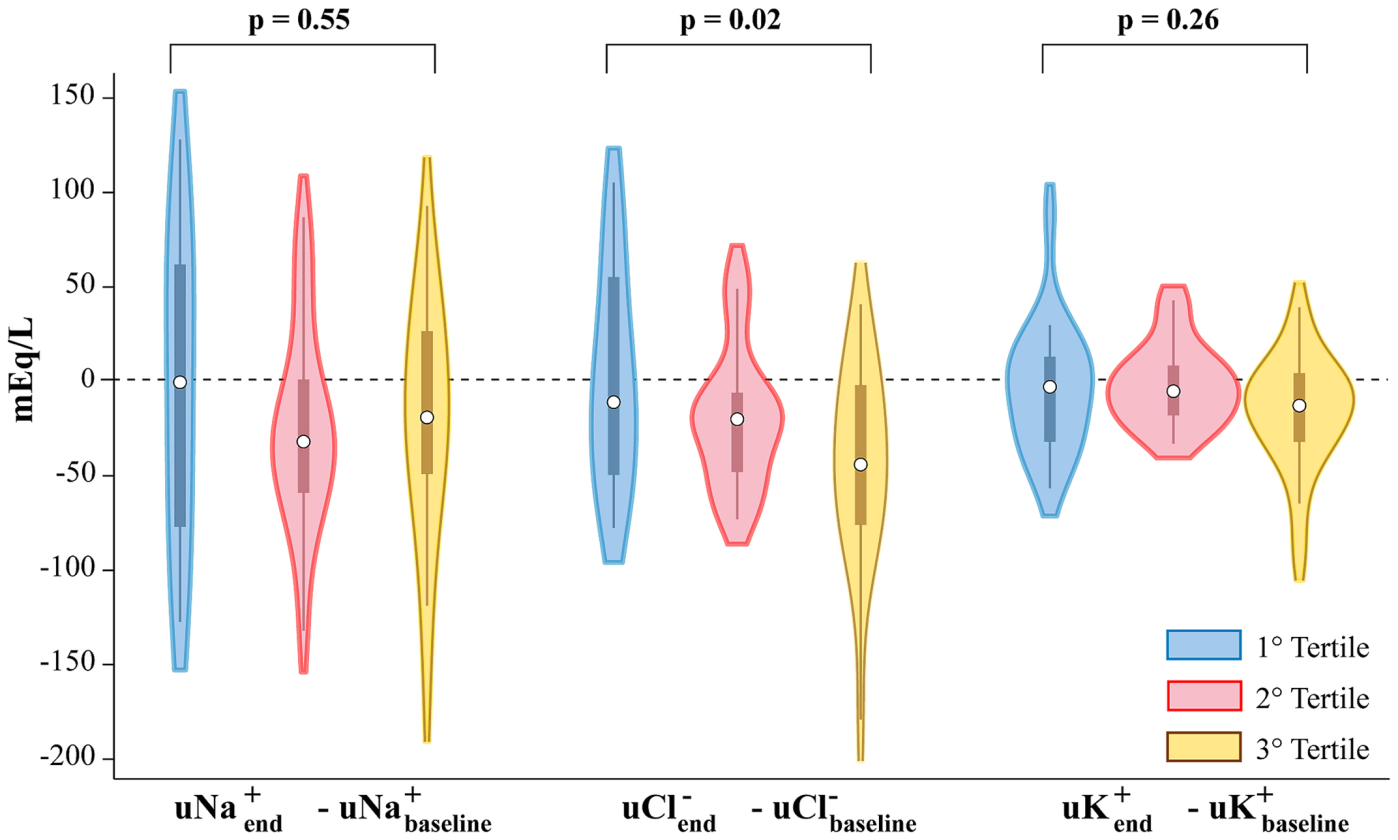
Difference between the end of the study and baseline urinary electrolytes, stratified by tertiles of the mean SID_INF_–HCO₃^−^ difference. Blue plots represent the first tertile, red plots the second tertile, and yellow plots the third tertile. Variations are calculated as the difference between end-of-study and baseline values for each parameter. Data are presented as violin plot (median reported by the white dot, 25th-75th percentile, whiskers indicate the 10th–90th percentiles). *p* values refer to one-way analysis of variance (ANOVA) performed for each electrolyte.

Overall, a negative, though quantitatively modest, association was observed between changes in SID_PL_ and mean uAG (Pearson’s *r* = −0.41, *p* = 0.001; Supplementary Figure S3). Moreover, no differences in mean uAG were observed according to the predominant crystalloid received (*p* = 0.87, Supplementary Table S4) or across SID_INF_-HCO_3_^−^ tertiles (*p* = 0.94, Table 5). Similarly, no differences in urinary excretion of Na^+^, K^+^, and Cl^−^ were observed during the study period in relation to SID_INF_ groups or SID_INF_-HCO_3_^−^ tertiles. When considering changes in urine electrolyte concentrations from single-spot samples obtained at baseline and at study end, uNa^+^ and uK^+^ remained unchanged over time regardless of the predominant crystalloid received. In contrast, changes in uCl^−^ differed significantly across SID_INF_ groups (*p* = 0.02), particularly in the Rehydrating-III group, where uCl^−^ decreased from 141 ± 49 to 103 ± 45 mEq/L (*p* < 0.001; Supplementary Table S5). Similar results were observed across SID_INF_-HCO_3_^−^ tertiles. In particular, ΔuCl^−^ decreased significantly across tertiles (Figure 3), consistent with progressive Cl^−^ retention during the study period, especially in the third SID_INF_-HCO_3_^−^ tertile (*p* < 0.001; Supplementary Table S6).

**Table 5:** Mean concentration and total amount of urinary electrolytes excreted during the study period, stratified according to the tertiles of the SID_INF_–HCO₃^−^ difference.

Variable	Overall population (*n* = 57)	T_1_ (*n* = 19) [−18.5–2.5]	T_2_ (*n* = 19) [2.6–18.5]	T_3_ (*n* = 19) [18.6–34.1]	*p*-value
Mean urinary specimen – mEq/L					
_U_Na^+^	120 ± 37	118 ± 34	125 ± 34	117 ± 43	0.80
_U_K^+^	59 ± 26	61 ± 24	56 ± 27	61 ± 28	0.78
_U_Cl^−^	126 ± 37	124 ± 43	129 ± 34	125 ± 36	0.92
_U_AG	53 ± 30	55 ± 29	52 ± 30	53 ± 32	0.94
_U_Na^+^ − _U_Cl^−^	−6 ± 20	−6 ± 20	−4 ± 16	−8 ± 23	0.83
Total excreted electrolytes – mEq					
_U_Na^+^	149 ± 92	135 ± 96	175 ± 111	136 ± 63	0.32
_U_K^+^	62 ± 24	57 ± 21	64 ± 31	64 ± 19	0.53
_U_Cl^−^	154 ± 94	140 ± 103	178 ± 113	144 ± 59	0.40

_u_Na^+^ denotes urinary sodium; _u_K^+^ urinary potassium; _u_Cl^−^ urinary chloride. Data are presented as mean ±standard deviation. *p* values refer to one-way analysis of variance (ANOVA), or Kruskal-Wallis one-way ANOVA on ranks as appropriate.

## Discussion

In this prospective observational study of ICU patients admitted after major surgery and receiving fluid therapy, we found that the greater the average SID_INF_ and SID_INF_-HCO_3_^−^ difference, the greater the increase in SID_PL_ and SBE after fluid administration, with minimal changes when the SID_INF_-HCO_3_^−^ difference approximated 0 mEq/L. Moreover, we observed that changes in unmeasured anions, reflected by SIG variations, contributed significantly to SBE changes. Finally, we found that fluid-induced acid–base changes were associated, within a short time frame, with a modulation of urinary Cl^−^ excretion, although this response did not quantitatively influence plasma acid–base status.

Overall, in this cohort of postoperative patients receiving fluid therapy with mild positive fluid balance, we found that the average SID_INF_ administered increased SID_PL_ and SBE to varying extents. The magnitude of these changes was primarily determined by the SID_INF_ value and by its difference with pre-infusion plasma HCO_3_^−^ concentration. Indeed, the greater the SID_INF_-HCO_3_^−^ difference, the greater the increase in SID_PL_ and SBE. Notably, in both the Sterofundin group and the first SID_INF_-HCO_3_^−^ tertile, where SID_INF_-HCO_3_^−^ difference approximated 0 mEq/L (−1.3 ± 12.3 and −3.8 ± 6.0 mEq/L, respectively), SID_PL_ remained unchanged or slightly decreased, and ΔSBE appeared lower than in other groups. Taken together, these findings further highlight the importance of considering pre-infusion HCO_3_^−^ concentration and its relationship with SID_INF_ to better describe fluid-induced changes in SID_PL_ and acid–base. In contrast to our previous investigation (15), pre-infusion HCO_3_^−^ concentrations showed limited variability (18.5–29.6 mmol/L), resulting in modest differences in acid–base and SID_PL_ responses across SID_INF_ and SID_INF_-HCO_3_^−^ subgroups. Nonetheless, the SID_INF_ value minimizing acid–base perturbations (≈24 mEq/L) closely matched baseline HCO₃^−^ concentration (≈23 mEq/L, see Supplementary Table S2), further supporting the integrative value of pre-infusion HCO₃^−^.

In contrast with our hypothesis, we did not observe significant A_TOT_ dilution after fluid administration. Accordingly, a key methodological premise underlying the integrative value of SID_INF_-HCO_3_^−^ in describing fluid-induced acid–base changes during A_TOT_ dilution (i.e., during fluid resuscitation or replacement) was not met. Indeed, whereas during fluid resuscitation or replacement the reference for SID_INF_ in predicting acid–base changes should be the pre-infusion HCO_3_^−^ concentration (≈24 mEq/L under normal conditions), during fluid maintenance it should, in theory, approximate SID_PL_ (≈40–42 mEq/L under normal conditions), as no A_TOT_ dilution occurs. Although A_TOT_ was not assessed in our previous investigation (15), the degree of hemodilution and volume expansion in the current study, as reflected by changes in hemoglobin (−0.5 ± 1.7 g/dL) and fluid balance (+802 ± 1,212 mL), was similar to that observed in our previous investigation (−0.7 ± 1.2 g/dL and +893 ± 1,296 mL, respectively), where, despite a similar design in a larger population, SID_INF_-HCO_3_^−^ appeared more informative than SID_INF_ alone in describing fluid-induced acid–base changes. Therefore, we cannot exclude that our sample size was underpowered to detect a small A_TOT_ dilution. Similarly, dynamic changes in A_TOT_ during the study period were not characterized, as measurements were available only at study entry (before fluid administration) and at study end (after fluid administration). It is therefore conceivable that variations in fluid administration rates over time resulted in different effects on A_TOT_ concentration, which could not be captured by our study design. Overall, our findings may lie between the two conceptual boundaries: a “fluid maintenance model”, characterized by unchanged A_TOT_ and SID_PL_ as the reference, and a “fluid resuscitation/replacement model” characterized by significant A_TOT_ variation and pre-infusion HCO_3_^−^ as reference. Further studies are warranted to define how the relative contribution of the different determinants of fluid-induced acid–base changes evolves over time across the continuum from fluid resuscitation to fluid maintenance.

During the study period, in addition to the effects of SID_INF_ on acid–base, we observed a mild, but significant, reduction in SIG (*Δ*-1.2 ± 2.4 mEq/L). These changes were largely independent of both the predominant crystalloid administered (SID_INF_) and the magnitude of the SID_INF_-HCO_3_^−^ difference (interaction *p* = 0.71 and 0.35, respectively; Tables 2, 3), suggesting a mechanism not directly related to fluid composition, assuming complete metabolism of the organic anions contained in the administered crystalloids (lactate, citrate, and acetate; see Supplementary Table S1). Within Stewart’s framework, SIG quantifies unmeasured anions and is defined as the difference between apparent SID (SID_PL_) and the effective SID, the latter corresponding to the sum of HCO_3_^−^ and A^−^, which account for the negative charges required to maintain electroneutrality (22, 27). The presence of unmeasured anions is common in critically ill patients, although their origins remain poorly characterized (27–30). In our study, although their specific nature was not assessed, their relatively high concentration at ICU admission may reflect a stress response or transient hypoxia-associated mitochondrial dysfunction related to surgery, with release of Krebs cycle intermediates, as recently reported (31). Although a dilutional effect cannot be excluded, these processes likely normalize early after ICU admission, independently of fluid composition. Regardless of mechanism, our findings highlight the importance of considering both the presence and temporal changes of unmeasured anions, even when small, in understanding acid–base alterations in this setting. Notably, SBE increased significantly despite unchanged SID_PL_ in both the Sterofundin and Miscellaneous groups and in the first SID_INF_-HCO_3_^−^ tertile, owing to a concomitant reduction in SIG.

Urinary electrolyte excretion showed no quantitative contribution to SID_PL_ or SBE changes over the study period, irrespective of fluid therapy. UAG was similar across SID_INF_ and SID_INF_-HCO_3_^−^ subgroups and was weakly associated with ΔSID_PL_ (slope coefficient −0.03 mEq/L). These findings suggest that, within the time window considered (~19 h), and the observed magnitude of acid–base alterations, the kidney does not meaningfully influence fluid-induced acid–base changes. A comparison of the volumes involved helps interpret these findings. Although measured in plasma, SID_PL_ reflects the extracellular space, which averages ~14–16 liters and may be expanded in postoperative critically ill patients (32). In our study, urine output was ~1.2 liters (range from 0.2 to 3.6 liters). If, for example, a 4 mEq/L decrease in plasma Cl^−^ were required during fluid therapy to maintain acid–base balance, and assuming no other relevant sources of Cl^−^ excretion, the kidney would need to excrete ~70 mEq of additional Cl^−^. This would require urine Cl^−^ to increase by >50 mEq/L from baseline values of ~140 mEq/L, approaching the physiological concentrating limits of the renal system (33, 34). This simple calculation highlights the marked disproportion between extracellular volume and urine output, limiting rapid modulation of SID_PL_ through urinary electrolyte excretion. Although we did not examine extreme acid–base disturbances, which may elicit a more pronounced renal response (35), our findings indicate that renal effects on plasma acid–base alterations require time to become quantitatively relevant, consistent with the time required for full renal compensation of respiratory acid–base disorders, which typically occurs over 3–5 days (36–38).

The lack of quantitative association between urinary electrolyte excretion and SID_PL_ changes over the study period should also be interpreted in light of the physiological meaning of uAG in the context of body electrolyte balance. UAG is defined as the difference between the urinary strong ions routinely measured in urine (Na^+^, K^+^, and Cl^−^) (25, 39). Based on the principle of electrical neutrality, it was originally proposed and is commonly used as a surrogate marker of urinary NH_4_^+^, the strong ion mainly responsible for unmeasured urinary cations and a key product of renal tubular acid–base handling (24, 40). However, when uAG is interpreted in relation to SID_PL_, an important physiological difference should be considered (16). While K^+^ is minimally represented in the extracellular space, being predominantly an intracellular cation, it is physiologically relevant in urine, where concentrations range from 25 to 200 mEq/L (17 to 124 mEq/L in our cohort) (34). Moreover, urinary K^+^ excretion depends not only on acid–base balance but also on extracellular K^+^ concentration, circulating volume sensing (RAAS activation), and extracellular K^+^ load from dietary and endogenous sources (e.g., intracellular release during cell lysis). Thus, while Na^+^, K^+^, and Cl^−^ in plasma mainly reflect the extracellular compartment, in urine they reflect processes involving both extracellular and intracellular compartments. Of note, when the study population was stratified by tertiles of average uAG, K^+^ was its only determinant, increasing markedly across tertiles (Supplementary Table S7).

Single spot urine analysis obtained at the beginning and end of the study provided additional information. Whereas uNa^+^ and uK^+^ remained unchanged across SID_INF_ and SID_INF_-HCO_3_^−^ subgroups, uCl^−^ significantly decreased in the Rehydrating-III group and across SID_INF_-HCO_3_^−^ tertiles, consistent with a progressive Cl^−^ retention. Notably, these were also the groups showing mild metabolic alkalosis, as reflected by SBE at study end. Although no further data on renal function were available, it is conceivable that the reduction in uCl^−^ reflects a physiological response of distal collecting tubules to metabolic alkalosis, with activation of the luminal Cl^−^/HCO_3_^−^ exchanger pendrin, leading to Cl^−^ reabsorption and HCO_3_^−^ excretion (41). Taken together, our findings suggest that although renal correction of plasma acid–base alterations requires time, the renal system responds rapidly and sensitively to these changes.

Our study also has some limitations. First, the degree of hemodilution and the amount of fluids administered were relatively limited, with modest net positive fluid balance. Nonetheless, the physiological principles described remain applicable within this clinical context. Second, acid–base variables were assessed at only two time points (ICU admission and study end), limiting assessment of the temporal dynamics of respiratory and renal responses and of changes in SID_PL_ and A_TOT_ dilution. In addition, patients were admitted after major surgery, when ongoing processes may have resulted in unstable baseline acid–base status. Third, the SID_INF_ of the administered crystalloids was consistently ≥20–24 mEq/L; accordingly, lower SID_INF_ values (e.g., normal saline) were not evaluated. Because normal saline is generally avoided at our center owing to its acid–base effects, this comparison was not feasible. Fourth, our analysis did not account for potential electrolyte redistribution related to albumin ion binding or transerythrocyte electrolyte shifts (42), although these processes are likely to become quantitatively relevant only at extreme pH values. Finally, electrolyte excretion was assessed only in urine and limited to Na^+^, K^+^, and Cl^−^, with no measurements from other excreted fluids.

## Conclusion

In conclusion, in postoperative critically ill patients receiving fluid replacement, the relationship between SID_INF_ and pre-infusion HCO_3_^−^ appears to be an important factor influencing fluid-induced acid–base changes, whereas changes in unmeasured anions appear to contribute independently to SBE variations. Despite modulation of urinary electrolyte excretion, renal effects do not appear to quantitatively influence plasma acid–base status over the short time frame studied.

## Data Availability

The raw data supporting the conclusions of this article will be made available by the authors, without undue reservation.
