# Supplementary material for: Fluid-induced acid–base variations in postoperative critically ill patients: physiological determinants and renal response
Source: Front Med (Lausanne). 2026 Jul 1;13:1867317. doi: 10.3389/fmed.2026.1867317 (PMC13369305; doi:10.3389/fmed.2026.1867317)

Fluid-induced acid-base variations in postoperative critically ill patients: physiological determinants and renal response

Francesco Zadek[1]*, Luca Zazzeron[2]*, Michele Ferrari[3], Davide Ottolina[4], Matteo Nafi[5], Floriana Ferrari[6], Eleonora Scotti[7], Federica Vagginelli[8], Marco Lattuada[9], Micah Liam Arthur Heldeweg[10,11], Lorenzo Giosa[11], Martin Krbec[11], Thomas Langer[1,3] and Pietro Caironi[12,13]

[1] Department of Medicine and Surgery, University of Milan-Bicocca, Monza, Italy;

[2] Department of Anesthesia, Critical Care and Pain Medicine, Massachusetts General Hospital, Boston, MA, USA;

[3] Department of Anesthesia and Intensive Care Medicine, Niguarda Ca' Granda, Milan, Italy;

[4] UO Anestesia e Rianimazione, Ospedale di Saronno, ASST Valle Olona, Saronno, Italy;

[5] Department of Anesthesia and Intensive Care Medicine, IRCCS Multimedica, Sesto San Giovanni, Italy;

[6] Pediatric Intensive Care Unit, Department of Anesthesiology and Intensive Care, ASST Papa Giovanni XXIII, Bergamo, Italy;

[7] Department of Anesthesia, Critical Care and Emergency, Fondazione IRCCS Ca' Granda-Ospedale Maggiore Policlinico, Milan, Italy;

[8] UO Anestesia e Rianimazione, Ospedale di Treviglio, ASST Bergamo Ovest, Treviglio, Italy;

[9] Anaesthesia and Intensive Care Unit, E.O. Ospedali Galliera, Genoa, Italy;

[10] Department of Anesthesiology, Amsterdam University Medical Centers, Boelelaan 1117, 1081HV, Amsterdam, The Netherlands;

[11] Department of Anaesthesiology and Intensive Care, 3rd Faculty of Medicine, Charles University and Kralovske Vinohrady University Hospital, Prague, Czech Republic;

[12] Department of Oncology, University of Turin, Turin, Italy;

[13] Department of Acute Brain and Cardiovascular Injury, Istituto di Ricerche Farmacologiche Mario Negri IRCCS, Milan, Italy.

* The two authors contributed equally to the present work.

ONLINE SUPPLEMENTARY MATERIALS

Supplementary tables

Table S1 – Composition of the main crystalloids administered during the study period.

	Sterofundin	Rehydrating III	Normal Saline (0.9% NaCl)	Ringer Lactate
**Na^{+} – mEq/L**	145	140	154	130
**K^{+} – mEq/L**	4	10	-	4
**Ca^{2+} – mEq/L**	5	5	-	3
**Mg^{2+} – mEq/L**	2	3	-	-
**Cl^{-} – mEq/L**	127	103	154	109
**Lactate – mmol/L**	-	-	-	28
**Citrate – mmol/L**	-	8	-	-
**Acetate – mmol/L**	24	47	-	-
**Osmolarity – mmol/L**	312	307	308	274
**In-vivo SID – mEq/L**	24	55	0	28

Na^{+} denotes sodium, K^{+} potassium, Ca^{2+} ionized calcium, Mg^{2+} magnesium, Cl^{-} chloride.

Table S2 – Blood gases, acid-base parameters, and electrolyte characteristics at baseline and end of the study.

Variable	Baseline (n = 57)	End of study (n = 57)	p-value
**pH**	7.41 ±0.05	7.42 ±0.03	0.11
**PCO_2 – mmHg**	36.7 ±5.0	39.6 ±4.4	<0.001
**HCO_3^- – mEq/L**	23.1 ±2.3	25.6 ±2.9	<0.001
**SBE – mmol/L**	-1.5 ±2.4	0.9 ±2.9	<0.001
**SID_{PL} – mEq/L**	38.7 ±2.4	39.8 ±2.7	<0.001
**SID_{EFF} – mEq/L**	33.7 ±3.0	36.0 ±3.6	<0.001
**SIG – mEq/L**	5.0 ±2.8	3.9 ±2.5	<0.001
**Na^+ – mEq/L**	138.1 ±2.8	138.7 ±2.7	0.08
**K^+ – mEq/L**	3.8 ±0.4	4.0 ±0.4	<0.001
**Ca^{2+} – mEq/L**	2.0 ±0.1	2.1 ±0.1	0.16
**Mg^{2+} – mEq/L**	1.5 ±0.2	1.6 ±0.2	<0.001
**Cl^- – mEq/L**	105.7 ±2.8	105.7 ±3.1	0.96
**Lac^- – mEq/L**	1.1 ±0.7	1.0 ±0.5	0.09
**A_{TOT} – mmol/L**	13.1 ±1.9	12.9 ±1.6	0.08
**Alb^- – mEq/L**	8.7 ±1.4	8.3 ±1.3	<0.001
**P^- – mEq/L**	1.8 ±0.4	2.1 ±0.4	<0.001
**PaO_2 – mmHg**	159 ±49	82 ±26	<0.001
**FiO_2 – %**	44 ±8	26 ±12	<0.001

**Hb – g/dL**	11.5 ±1.6	11.0 ±1.6	0.006

$PaCO_2$ denotes arterial partial pressure of carbon dioxide; HCO_3^- bicarbonate concentration; SBE standard base excess; SID_{PL} plasma strong ion difference; SID_{EFF} effective strong ion difference; SIG strong ion gap; A_{TOT} total concentration of non-carbonic weak acids; Alb^- ionized concentration of albumin; P^- ionized concentration of phosphate; Hb hemoglobin; PaO_2 arterial partial pressure of oxygen; and FiO_2 fraction of inspired oxygen. Data are presented as mean ±standard deviation. P values refer to the paired t-test of the Wilcoxon signed rank test as appropriate.

Table S3 – Fluid shift and electrolytes received during the ICU stay.

Characteristics	Overall Population (n = 57)	T_1 (n = 19)	T_2 (n = 19)	T_3 (n = 19)	p-value
**Infused SID - HCO_3^-**		-3.8 ±6.0	12.1 ±4.3	23.5 ±4.7	< 0.001
**Total amount of administered fluids – mL**	3152 ±1027	3366 ±1150	2932 ±972	3159 ±955	0.44
**Infused Solutions – mL**					
**Sterofundin**	580 ±1086	1554 ±1372	134 ±478	53 ±229	<0.001
**Normal Saline**	362 ±728	690 ±993	292 ±603	104 ±329*	0.04
**Rehydrating III**	1412 ±1071	326 ±558	1634 ±626	2276 ±883	<0.001
**Others[a]**	417 ±369	401 ±443	459 ±327	392 ±344	0.84
**Fluid Creep**	381 ±141	395 ±123	413 ±141	334 ±152	0.20
**Infused Electrolytesb – mEq**					
**Na^+**	422 ±145	456 ±151	386 ±140	424 ±143	0.33
**K^+**	34 ±24	21 ±22	41 ±20	41 ±25	0.01
**Ca^{2+}**	11 ±5	11 ±7	11 ±4	12 ±5	0.58
**Mg^{2+}**	9 ±5	6 ±5	8 ±4*	12 ±6*	0.004
**Cl^-**	371 ±132	432 ±136	341 ±131	340 ±112	0.046
**Total amount of excreted fluids – mL**	2350 ±693	2134 ±663	2545 ±855	2372 ±485	0.19
**Drainage**	397 ±380	314 ±232	439 ±511	440 ±353	0.51
**Nasogastric Losses**	57 ±127	72 ±128	72 ±169	28 ±62	0.47

**Insensible Perspiration**	699 ±173	652 ±142	700 ±197	745 ±173	0.26
**Diuresis**	1197 ±612	1096 ±707	1334 ±719	1160 ±343	0.47
**Net fluid balance – mL**	802 ±1212	1232 ±1360	387 ±1246	787 ±889	0.10
**Diuretic – n. (%)**	21 (37)	6 (32)	8 (42)	7 (37)	0.94
**Infused SID – mEq/L**	33.7 ±13.1	18.2 ±5.5	36.0 ±6.0 *	46.8 ±5.0*	<0.001
**HCO_3^- – mEq/L**	23.1 ±2.3	22.1 ±1.7	23.9±2.2*	23.4 ±2.7	0.04

Fluid therapy and electrolytes administered during the ICU stay, stratified according to the tertiles of the SID_{INF}–HCO_3^- difference. Na^+ denotes sodium; K^+ potassium; Ca^{2+} ionized calcium; Mg^{2+} magnesium; Cl^- chloride; SID_{INF} infused SID. Data are presented as mean ±standard deviation or n (%), as appropriate. P values refer to one-way analysis of variance (ANOVA), Kruskal-Wallis one-way ANOVA on ranks, or chi-square test as appropriate.
* = p< 0.05 vs first tertile; a = "others" encompasses the administered volumes of other crystalloids, colloids, drugs, and electrolytes correction; b = the infused electrolytes balance account also for the presence of additional electrolytes supplementation.

Table S4 – Mean urinary specimen electrolytes divided by infused solution.

	Overall	Sterofundin	Miscellaneous	Rehydrating III	p-value
**Mean urinary specimen – mEq/L**					
$_{U}Na^{+}$	120 ±37	123 ±33	115 ±37	123 ±40	0.72
$_{U}K^{+}$	59 ±26	53 ±20	69 ±30	54 ±23	0.09
$_{U}Cl^{-}$	126 ±37	125 ±38	128 ±42	125 ±34	0.97
$_{U}AG$	53 ±30	52 ±21	56 ±36	52 ±30	0.87
$_{U}Na^{+}$ - $_{U}Cl^{-}$	-6 ±20	-2 ±11	-13 ±21	-2 ±21	0.13
**Total excreted electrolytes – mEq**					
$_{U}Na^{+}$	149 ±92	160 ±100	137 ±114	153 ±69	0.75
$_{U}K^{+}$	62 ±24	61 ±21	62 ±31	62 ±20	0.98
$_{U}Cl^{-}$	154 ±94	163 ±107	148 ±120	154 ±62	0.91

Table S5 – Baseline and end of study urinary electrolytes of the population divided by infused solution.

Characteristics	Sterofundin (n = 13)	Miscellaneous (n = 20)	Rehydrating III (n = 24)	Group factor p-value	Interaction p-value
**$_U Na^+$ – mEq/L**					
**Baseline**	118 ±51	118 ±49	115 ±46	0.68	0.74
**End of study**	115 ±62	101 ±52	94 ±50		
**Time Factor p-value**		0.14			
**$_U K^+$ – mEq/L**					
**Baseline**	66.2 ±30	52 ±19	53 ±23	0.08	0.76
**End of study**	56.4 ±18	49 ±27	44 ±19		
**Time Factor p-value**		0.07			
**$_U Cl^-$ – mEq/L**					
**Baseline**	131 ±32	146 ±59	141 ±49	0.35	0.08
**End of study**	129 ±47	135 ±52	103 ±45*		
**Time Factor p-value**		0.02			
**$_U AG$ – mEq/L**					
**Baseline**	53 ±27	24±39	27±34	0.07	0.13
**End of study**	42 ±26	15 ±45	35±38		
**Time Factor p-value**		0.37			
**$_U Na^+$- $_U Cl^-$ – mEq/L**					
**Baseline**	-14 ±40	-28 ±46	-26 ±39		

**End of study**	-15 ±22	-34 ±37	-8 ±36	0.22	0.22
**Time Factor p-value**		0.59			

$_{u}Na^{+}$ denotes urinary sodium; $_{u}K^{+}$ urinary potassium; $_{u}Cl^{-}$ urinary chloride. Data are presented as mean ±standard deviation. P values refer to two-way analysis of variance (ANOVA) with post-hoc all pairwise multiple comparison procedures (Holm-Sidak correction methods). * = $p< 0.05$ vs baseline of the same solution

Table S6 – Baseline and end of study urinary electrolytes of the population, stratified according to the tertiles of the $SID_{INF}–HCO_3^-$ difference.

Characteristics	Overall Population (n = 57)	T_1 (n = 19) [-18.5 – 2.5]	T_2 (n = 19) [2.6 – 18.5]	T_3 (n = 19) [18.6 –34.1]	Group Factor p-value	Interaction p-value
**$_UNa^+$ – mEq/L**						
**Baseline**	117 ±47	125 ±50	115 ±46	111 ±47	0.34	0.94
**End of study**	101 ±53	113 ±61	100 ±51	91 ±46		
**Time Factor p-value**			0.09			
**$_UK^+$ – mEq/L**						
**Baseline**	56 ±24	59 ±25	52 ±24	56 ±22	0.07	0.45
**End of study**	48 ±22	57 ±24	45 ±18	42 ±21		
**Time Factor p-value**			0.16			
**$_UCl^-$ – mEq/L**						
**Baseline**	140 ±49	139 ±43	130 ±46	152 ±58	0.68	0.07
**End of study**	120 ±49	132 ±52	117 ±49	110 ±47*		
**Time Factor p-value**			0.003			
**$_UAG$ – mEq/L**						
**Baseline**	32 ±36	45 ±36	37 ±27	14 ±39	0.11	0.17
**End of study**	30 ±39	38 ±40	28 ±43	23 ±36		
**Time Factor p-value**			0.59			
**$_UNa^+$- $_UCl^-$ – mEq/L**						

**Baseline**	-24 ±42	-14 ±42	-15 ±31	-42 ±47	0.29	0.12
**End of study**	-19 ±35	-19 ±28	-18 ±42	-19 ±35*		
**Time Factor p-value**			0.42			

$_uNa^+$ denotes urinary sodium; $_uK^+$ urinary potassium; $_uCl^-$ urinary chloride. Data are presented as mean ±standard deviation. P values refer to two-way analysis of variance (ANOVA) with post-hoc all pairwise multiple comparison procedures (Holm-Sidak correction methods). * = p< 0.05 vs baseline of the same tertile

Table S7 – Mean urinary electrolytes excreted during the study period, stratified according to the tertiles of urinary anion gap (uAG).

Variables	Overall Population (n = 57)	T_1 (n = 19)	T_2 (n = 19)	T_3 (n = 19)	p-value
**$_U$AG – mEq/L**	53.3 ±30.0	20.7 ±9.1	52.3 ±11.3	86.8 ±16.6	<0.001
**Infused fluids – mL**					
**Normal Saline**	362 ±728	488 ±962	263 ±639	335 ±535	0.63
**Sterofundin**	580 ±1086	383±907	1127 ±1363	230.7 ±708	0.02
**Rehydrating III**	1412 ±1071	1488 ±1006	928 ±1069	1820 ±992	0.03
**Diuresis – mL**	1197 ±612	1494 ±855	1079 ±311	1017 ±443	0.03
**Diuretic therapy – n. (%)**	21 (37)	14 (74)	3 (16)	4 (21)	<0.001
**Fluid balance – mL**	802 ±1212	628 ±1295	991 ±1235	787 ±1139	0.66
**Mean urinary specimen – mEq/L**					
**$_U$Na$^+$**	120.0 ±36.8	116.3 ±30.7	118.2 ±43.6	125.5 ±36.4	0.73
**$_U$K$^+$**	59.1 ±25.9	35.7 ±17.5	59.8 ±15.1	81.8 ±20.8	<0.001
**$_U$Cl$^-$**	125.8 ±37.3	131.2 ±35.2	125.6 ±44.1	120.5 ±32.8	0.68
**$_U$Na$^+$- $_U$Cl$^-$ – mEq/L**	-5.8 ±19.9	-14.9 ±16.0	-7.5 ±10.5	5.0 ±25.0	0.02

Data are presented as mean ±standard deviation or n (%), as appropriate. P values refer to one-way analysis of variance (ANOVA), or Kruskal-Wallis one-way ANOVA on ranks as appropriate.

Figure S1. Frequency distribution of patients according to the SID infused (SID_{INF}) during the study period.

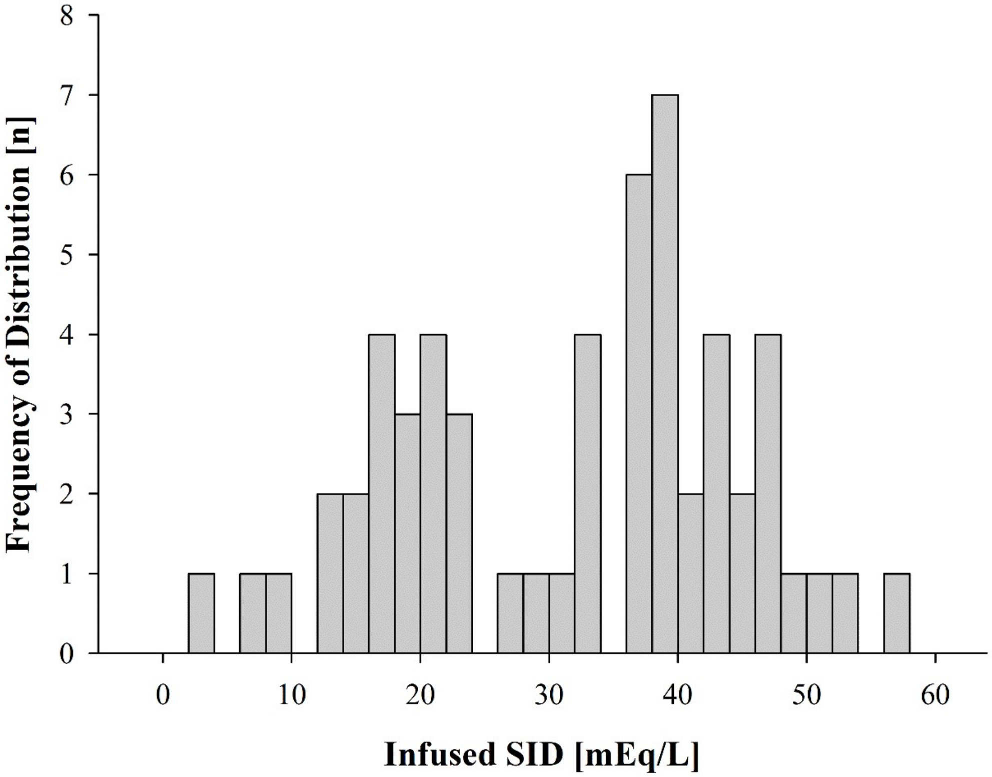

For each patient, the average SID_{INF} was calculated considering all the sources of fluid administered during the study period (see method section of the main manuscript for further details).

Figure S2. Frequency distribution of patients according to the pre-infusion plasma HCO_3^- concentration recorded at study baseline.

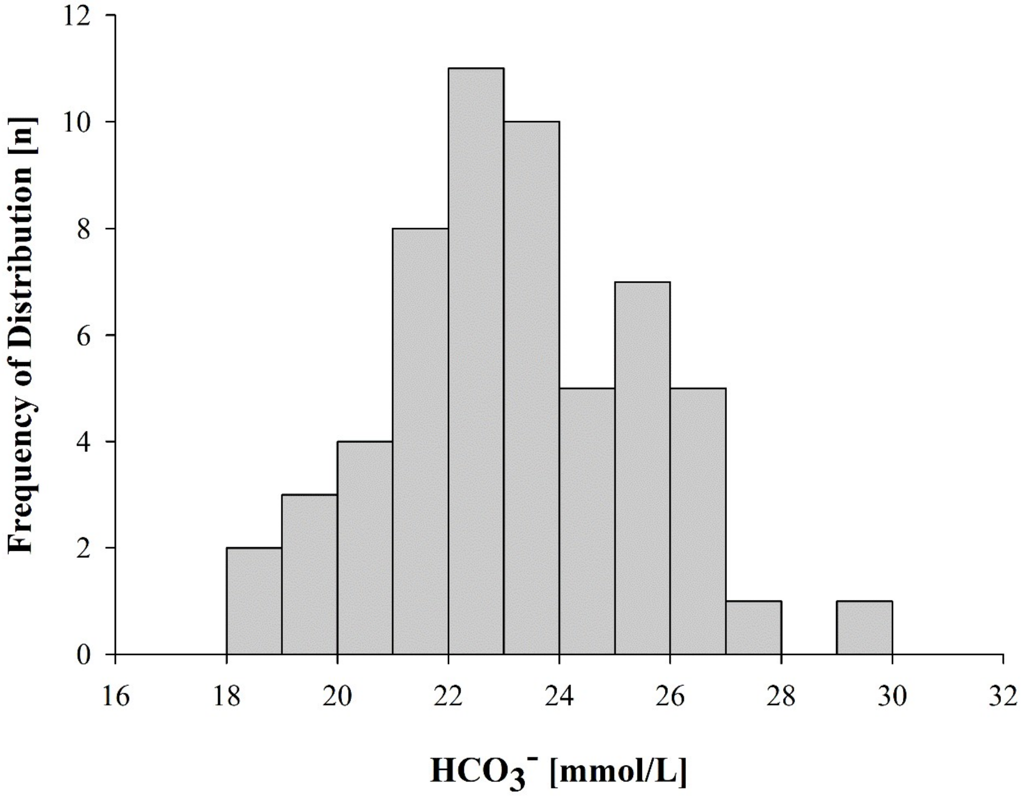

Figure S3. Association between the average variation of plasma SID (ΔSID) and the mean urinary anion gap (uAG) excreted during the study period. Pearson's r =-0.41, p=0.001. Model's equation: $\Delta SID = -0.03 \cdot uAG + 2.91$

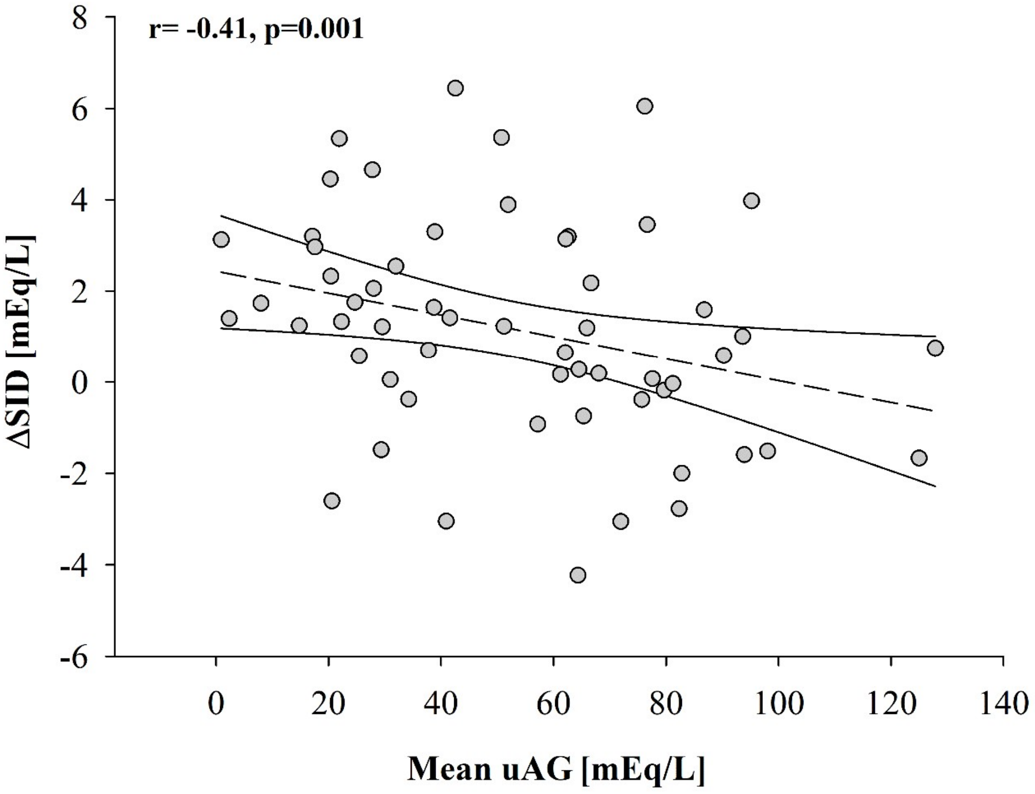

Supplement: Supplementary file 1 [file Supplementary_file_1.pdf]
